# Metabolic Comparison of Mature and Immature Bovine Cumulus–Oocyte Complexes with Standardization of Bioenergetic Assessment

**DOI:** 10.3390/biom16020317

**Published:** 2026-02-18

**Authors:** Cristina Algieri, Emilia Attolini, Eleonora Iacono, Salvatore Nesci, Barbara Merlo

**Affiliations:** 1 Department of Veterinary Medical Sciences, University of Bologna, 40064 Bologna, Italy; cristina.algieri2@unibo.it (C.A.); emilia.attolini2@unibo.it (E.A.); eleonora.iacono2@unibo.it (E.I.); barbara.merlo@unibo.it (B.M.); 2 Department of Life Sciences, Health and Health Professions, Link University, 00165 Rome, Italy

**Keywords:** cumulus-oocyte complex, IVM, cell metabolism, bioenergetic analysis, ATP production

## Abstract

This study compared the bioenergetic profiles of immature and in vitro–matured bovine cumulus–oocyte complexes (COCs) using Seahorse extracellular flux technology, with the aim of establishing standardized conditions for real-time metabolic assessment during in vitro maturation (IVM). Groups of five COCs were analysed prior to maturation and after 22 h of IVM using the Seahorse XFp Analyzer to measure oxygen consumption rate (OCR, pmoL/min) and extracellular acidification rate (ECAR, mpH/min), providing dynamic readouts of oxidative phosphorylation and glycolysis that extend beyond conventional endpoint assays. To optimize assay performance, three media were first evaluated: TCM199, DMEM/F12, and HEPES-buffered synthetic oviductal fluid (HSOF). HSOF yielded the most reliable readings for immature COCs, whereas TCM199 provided superior conditions for mature COCs. Adhesion strategies were then tested by comparing uncoated wells with wells coated with fibronectin, concanavalin A, or Matrigel^®^. Sequential injections of oligomycin and rotenone plus antimycin A enabled partitioning of mitochondrial and glycolytic contributions to ATP production. COC maturation was associated with a clear metabolic shift from glycolysis toward oxidative metabolism. Immature COCs displayed a predominantly glycolytic phenotype, while mature COCs showed increased active mitochondrial ATP production. Adhesion conditions markedly affected the detected metabolic profile: concanavalin A and fibronectin supported effective attachment and were associated with robust energy metabolism, whereas Matrigel^®^ and poor adhesion were linked to quiescent profiles with low OCR and ECAR signals. Together, these data define practical assay parameters for extracellular flux analysis of COCs and highlight the increasing reliance on mitochondrial function as a hallmark of oocyte maturation, supporting improved metabolic phenotyping for IVM optimization.

## 1. Introduction

Metabolism plays a central role in determining oocyte quality. Cumulus expansion, meiotic resumption, and advancement to metaphase II (MII) stage occur rapidly and are highly energy-dependent, requiring tightly regulated metabolic support [1]. Mitochondria represent one of the most prevalent organelles in oocytes [2], and their activity is considered essential for proper maturation and early embryonic development. Mitochondrial distribution, morphology, and membrane potential dynamically change during oocyte growth and maturation, reflecting the oocyte’s energetic requirements at each developmental stage. Dysfunctional mitochondria or insufficient mitochondrial biogenesis have been associated with reduced ATP production, increased reactive oxygen species (ROS), and impaired embryonic competence, highlighting the importance of mitochondrial integrity for reproductive success [3].

In mammals, the growth and maturation of the oocyte are tightly coordinated with the activity of surrounding somatic cells, particularly cumulus cells. Together, they form the cumulus–oocyte complex (COC), a structurally and functionally specialized unit. This association is maintained through intercellular membrane channels, called gap junctions, that allow direct communication and metabolic exchange between the oocyte and cumulus cells [4]. Gap junction-mediated communication is bidirectional, allowing the oocyte to receive essential metabolites and signalling molecules while simultaneously influencing cumulus cell gene expression and metabolic activity. Disruption of these channels during IVM or pathological conditions can lead to altered nutrient flux, impaired energy production, and reduced oocyte quality, emphasizing the delicate interplay between structural connectivity and metabolic function within the COC [5].

There is a metabolic cooperation within the COC. Cumulus cells play a pivotal role in maintaining oocyte ATP levels by supplying metabolic intermediates via gap junctions, while the oocyte actively influences cumulus cell metabolism by modulating their gene expression through the secretion of soluble growth factors that act directly on cumulus cells [6]. Glucose plays a crucial role in both cytoplasmic and nuclear maturation within the COC by fueling pyruvate production, which is essential for oocyte meiosis progression to the MII stage [7,8]. The oocyte itself has limited capacity to transport and metabolize glucose, so it relies on cumulus cells to perform glycolysis and supply pyruvate, which is then used by the oocyte’s mitochondria for ATP production via oxidative phosphorylation (OXPHOS). Oocyte metabolism differs depending on species and the stage of meiotic maturation. Most of the glucose consumed by bovine COCs at the onset of in vitro maturation (IVM) is primarily metabolized through glycolysis [9]. As maturation progresses, glucose metabolism shifts toward additional pathways to meet the evolving metabolic demands of the COC. Reduced glycolytic activity has been linked to poor oocyte developmental competence [10], while enhanced glycolysis correlates with improved blastocyst formation rates [11]. Furthermore, metabolic flexibility, the ability of the COC to shift between glycolysis, oxidative phosphorylation, and alternative pathways such as the pentose phosphate pathway, appears critical for meeting changing energy demands during maturation. Metabolic plasticity not only ensures sufficient ATP production but also contributes to redox homeostasis, biosynthesis of nucleotides, and maintenance of cellular signalling pathways essential for meiotic progression [12].

Early studies in murine oocytes suggested that ATP levels remain stable throughout maturation [13]. However, later research in bovine and porcine models revealed that ATP levels are significantly higher at the MII stage compared to the germinal vesicle (GV) stage [14,15,16]. Furthermore, COCs matured in vitro often exhibit metabolic profiles distinct from those matured in vivo. For instance, porcine and bovine oocytes undergoing IVM display lower glucose metabolism, reduced concentrations of the antioxidant glutathione, altered redox status, a decreased ATP/ADP ratio, and an accumulation of intracellular lipids, suggesting altered energy metabolism compared to in vivo conditions [11,17,18].

Understanding the metabolic activity of the intact COC is critical for optimizing IVM protocols and improving developmental outcomes. The oxygen consumption rate (OCR), a key indicator of oxidative metabolism, correlates with important reproductive parameters such as oocyte maturation, embryo morphology, implantation potential, and pregnancy outcomes [19]. However, direct investigations into COC metabolism remain limited, partly due to the technical complexity. This is largely because oocyte metabolism is not static, but it undergoes dynamic, stage-specific changes throughout maturation [20,21]. Thus, real-time measurement of metabolic activity at defined time points is likely to offer more informative insights than traditional endpoint assays. In this context, the Seahorse XFp Analyzer (Agilent, Santa Clara, CA, USA) emerges as a powerful and innovative tool that enables real-time, non-invasive analysis of cellular metabolism across multiple experimental conditions simultaneously [22]. It provides a comprehensive characterization of both glycolytic and oxidative activity by measuring OCR, reflecting mitochondrial respiration, and extracellular acidification rate (ECAR), a marker of glycolysis [23]. This dual assessment allows for a more detailed and dynamic understanding of the balance between energy-producing pathways within the COC [24]. By integrating OCR and ECAR measurements, it is possible to quantify not only absolute metabolic activity but also the relative contributions of glycolysis and mitochondrial respiration under different experimental conditions. This approach facilitates the identification of metabolic signatures predictive of oocyte competence, providing a valuable framework for standardizing IVM protocols and potentially improving embryo yield and quality in both livestock and clinical settings.

Therefore, this study aims to standardize a protocol for analysing bovine oocytes metabolism before and after maturation using the Seahorse Analyzer to better characterize oocyte bioenergetics.

## 2. Materials and Methods

### 2.1. Reagents

Seahorse XF Assay Kits and Reagents were purchased from Agilent. All chemicals were obtained from Sigma-Aldrich (Milano, Italy) unless otherwise stated.

### 2.2. Experimental Design

To identify differences in the metabolism of immature and mature bovine oocytes, groups of five COCs were analysed using Agilent Seahorse XFp analyzer (Agilent, Santa Clara, CA, USA) before maturation and after 22 h of IVM. Three different media, commonly used in in vitro embryo production (IVEP), were tested to determine the most suitable medium for COCs analysis: TCM199 (Tissue Culture Medium, Sigma M3769 supplemented with 10 mM glycine, 10 mM HEPES, and 26 mM NaHCO_3_), DMEM (Dulbecco’s Modified Eagle Medium, Agilent 103575) and an in-house prepared HSOF (modified synthetic oviductal fluid [25] without glucose, sodium pyruvate, and antibiotics and supplemented with 10 mM glycin, 10 mM HEPES, essential and non-essential amino acids, and 6 mg/mL fraction-V bovine serum albumin; 280–290 mOSm, pH 7.4). Once the optimal medium was identified, COCs were analysed on either uncoated wells or wells coated with one of three different materials: fibronectin, concanavalin A or Matrigel^®^. Each condition was tested in triplicate. Since concanavalin A had never been previously used as a coating substrate for COCs, its possible effects on bovine COC maturation were evaluated to exclude any influence on the meiotic progression. The comparison of different media was performed exclusively to optimize Seahorse assay performance and establish standardized conditions for metabolic measurements, and not to evaluate their suitability as in vitro maturation media.

### 2.3. Oocyte Collection and In Vitro Maturation

COCs were collected by aspirating 2–8 mm follicles from abattoir-derived ovaries using a 21 G butterfly needle connected to a 50 mL Falcon tube through a silicone plug. This setup was attached to a vacuum pump (Cook Italia Srl, Nova Milanese, Italy) set to −50 mmHg. Under a stereomicroscope, compact COCs exhibiting at least 4–5 layers of cumulus cells and a homogeneous, finely granulated cytoplasm were identified, selected, and subsequently washed twice in HSOF. Some oocytes were washed in Seahorse medium (TCM199, DMEM/F12, HSOF) and immediately analyzed, while others underwent IVM. The basal maturation medium consisted of TCM199 supplemented with 10 ng/mL epidermal growth factor (EGF), 100 ng/mL insulin-like growth factor-1 (IGF-1), 1.2 mM L-cysteine, 1 mM sodium pyruvate, 75 μg/mL kanamycin, 25 μL/mL Insulin-Transferrin-Sodium Selenite Supplement (ITS) and 0.1 IU/mL porcine FSH-LH (Pluset, Calier, Como, Italy). Groups of 5 COCs were matured in 100 µL drops of maturation medium under mineral oil for 22 h at 38.5 °C in humidified air at 5% CO_2_. After IVM, mature COCs were washed in Seahorse medium and then subjected to analysis.

### 2.4. Coating Testing

Concanavalin A was added to the IVM medium at the same concentration (0.39 mg/mL) and for the same exposure time (1 h) as used in the wells XFp cell culture mini-plates (Agilent, Santa Clara, CA, USA) for metabolic assessment. The test was performed in three independent replicates, with approximately 30 oocytes per group. Following aspiration of follicles and the selection of COCs, they were randomly assigned to two groups for IVM: (i) control group (CTR), COCs cultured in basal maturation medium for 22 h; (ii) concanavalin A group (ConA), COCs cultured in basal medium supplemented with concanavalin A for 1 h and then in basal medium for 21 h. After maturation, expanded COCs were gently pipetted to mechanically remove cumulus cells. Denuded oocytes were stained with 10 μg/mL of bisbenzimide (Hoechst 33342) in phosphate-buffered saline supplemented with 0.1% polyvinyl alcohol (PBS + PVA) for 15 min at room temperature in the dark. Oocytes were then washed in PBS + PVA, mounted on glass slides, covered with a coverslip, and examined under an epifluorescence microscope (Eclipse E, Nikon, Rome, Italy), equipped with a UV-2A (330–380 nm) excitation filter, to assess the meiotic stage. Oocytes were classified as mature (presence of an extruded polar body and a metaphase plate), immature (nuclear configurations ranging from the germinal vesicle stage to metaphase I), or degenerate (deteriorated or non-visible nuclear material).

### 2.5. COCs Metabolism Analysis

Approximately 5 COCs in 180 μL of medium were placed in each well of XFp cell culture mini-plates (Agilent, Santa Clara, CA, USA), previously uncoated and coated with 10 μL fibronectin (1 mg/mL in water), 10 μL concanavalin A (0.39 mg/mL in water), dried in an incubator at 37 °C for 2 h, and 3 μL Matrigel^®^ (1:1 in DMEM) left to solidify at room temperature for 30 min. Different media were previously tested and selected: HSOF for the best detection of metabolism of immature COCs and basal maturation medium for mature COCs, each one implemented with glucose 10 mM, pyruvate 1 mM and glutamine 2 mM, all preheated to 37 °C. Cellular respiration, as oxygen consumption rate (OCR) (pmoL/min) and extracellular acidification rate (ECAR), an index of glycolysis (mpH/min), was recorded using the Seahorse XFp (Agilent, Santa Clara, CA, USA) [26].

The injection ports of the XFp sensor cartridges were previously hydrated overnight at 37 °C with XF Calibrant and, shortly before starting the assay, loaded with a concentration of mitochondrial modulators ten times higher than that indicated in the Seahorse XFp ATP Rate assay instructions. Final concentrations of 1.5 μM oligomycin (olig, port A) and 0.5 μM rotenone (rot) plus 0.5 μM antimycin A (AA, port B) were used and injected serially, allowing calculation of mitochondrial and glycolytic ATP production rates and providing a real-time measurement of cellular ATP production rates. Data were analyzed using WAVE software 2.6.4.24 (Agilent, Santa Clara, CA, USA). The ATP rate assay is a fundamental test for evaluating cellular bioenergetics and is currently used to characterize ATP production in cells. Specifically, this approach allows for the distinct quantification of the contribution of the two main metabolic pathways involved in energy generation: glycolysis and mitochondrial OXPHOS.

The rate of ATP production from glycolysis (glycoATP) is closely related to the conversion of glucose to lactate, a process typically associated with conditions of rapid high energy demand or hypoxic states. This mechanism, while less efficient in terms of energy yield, allows for rapid and oxygen-independent ATP production, which is particularly relevant in highly proliferative or metabolically reprogrammed cells, such as tumor cells.

Likewise, the rate of mitochondrial ATP production (mitoATP) reflects the activity of the electron transport chain and OXPHOS, a highly efficient ATP-producing process dependent on oxygen availability. Oxygen-dependent processes are utilized primarily by differentiated and metabolically quiescent cells, where energy efficiency and the maintenance of cellular homeostasis take priority over the rate of ATP production.

The ratio of mitoATP production to glycoATP production, known as the ATP rate index, is therefore a valuable quantitative parameter for assessing the cellular metabolic phenotype. A ratio greater than 1 indicates a predominance of OXPHOS suggesting a primarily oxidative and mitochondria-dependent metabolism. Conversely, a ratio less than 1 indicates a greater reliance on glycolysis, reflecting a predominantly glycolytic metabolic structure. The combined analysis of glycoATP and mitoATP production rates provides a thorough and quantitative description of the cell’s energy strategy, significantly contributing to our understanding of metabolic adaptation mechanisms. Within the COCs, it reflects not only the metabolic state of individual cellular components, but also the degree of communication and metabolic cooperation between the oocyte and cumulus cells.

### 2.6. Statistical Analysis

Data represent the mean ± SD (shown as error bars in the figures) of three independent experiments. For IVM data, statistical analysis was conducted by 2 × 2 contingency table Chi-Square test (*p* < 0.05) using Statistics for Data Analysis v. 30 (SPS S.r.l., Italy). For metabolic data, statistical analysis was performed by ANOVA followed by Tukey’s multiple comparisons test (*p* ≤ 0.05) with GraphPad Prism software 11.0 (Adalta, Italy).

## 3. Results

### 3.1. Concanavalin a Effect on IVM

**Figure 1:**
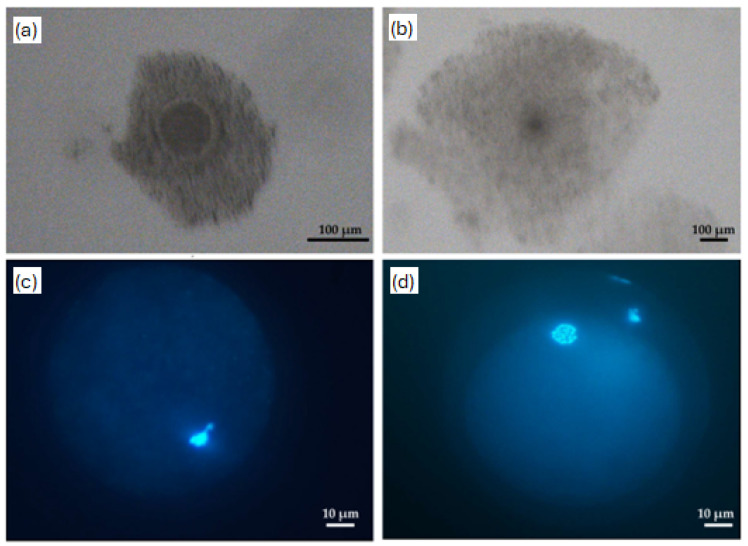
Representative images of bovine cumulus–oocyte complexes (COCs) before and after in vitro maturation (IVM) and nuclear status of immature and mature oocytes. Bright-field images show COCs at: (**a**) the immature stage (before IVM); (**b**) after 22 h of IVM, highlighting cumulus expansion following maturation. Representative epifluorescence images of denuded oocytes stained with Hoechst 33342 illustrate nuclear configurations of: (**c**) immature oocyte (germinal vesicle); (**d**) mature oocyte (metaphase II with extruded polar body).

A total of 172 oocytes were examined (Figure 1). The maturation rate was not affected by the exposure of bovine immature COCs to concanavalin A for 1 h (*p* > 0.05) (Table 1).

### 3.2. ATP Production in Immature COCs

**Figure 2:**
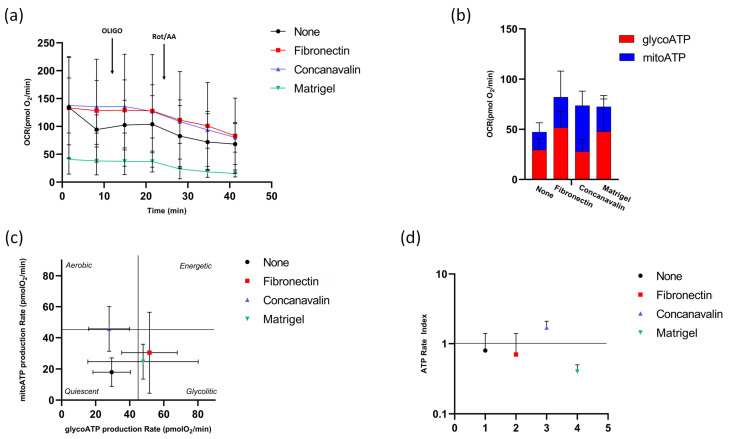
ATP production in immature COCs. (**a**) Metabolic profile obtained from oxygen consumption rate (OCR) in immature COCs, without (None) and with fibronectin, concanavalin and Matrigel^®^ under basal respiration conditions and after addition of 1.5 μM oligomycin (olig), and a mixture of 0.5 μM rotenone plus antimycin A (Rot/AA). Modulator injections are shown with arrows. (**b**) Evaluation of ATP production rate by mitochondrial OXPHOS (blue, mitoATP) or by glycolysis (red, glycoATP) in the different conditions. (**c**) Energy map of mitoATP Production Rate vs. glycoATP Production Rate in immature COCs without (black) and with fibronectin (red), concanavalin A (blue) and Matrigel^®^ (green). (**d**) The ratio between the mitochondrial ATP production rate and the glycolytic ATP production rate (ATP rate index) is shown on the *y*-axis (logarithmic scale) in immature COCs under different conditions.

The levels of ATP produced by OXPHOS and glycolysis in immature COCs were assessed in the presence of fibronectin, concanavalin A, and Matrigel^®^, as matrices for COC adhesion to the bottom of the plate, and compared to the condition without matrix (None), which are shown in Figure 2. The metabolic profile in the presence of different substrate coating for oocyte adhesion in Figure 1a was obtained following the serial addition of oligomycin, to inhibit mitochondrial ATP synthesis, and the mixture of rotenone and antimycin A, to block mitochondrial respiration. The results showed that the immature stage COCs did not respond to the effect of the oligomycin inhibitor, while they were sensitive to rotenone/antimycin A. The rate of mitoATP and glycoATP production showed that immature COCs relied predominantly on glycolysis for energy production under most experimental conditions, in contrast to cells on concanavalin-coated microplate, exhibit a shift in metabolic preference, with approximately 65% of total ATP production being supplied by mitochondria rather than by glycolysis (Figure 2b). The amount of total ATP recorded, calculated as the sum of mitoATP and glycoATP, appeared slightly higher when cells were coated at the bottom of the plate, although there was no significant difference between the various conditions (Figure 2b) (*p* > 0.05).

Figure 2c showed mitoATP production rate vs. glycoATP production rate during basal respiration, providing a quick overview of the metabolic state of the cells under different experimental conditions. A quiescent state of COCs tending towards a glycolytic pathway was evident in the absence and in the presence of fibronectin and Matrigel^®^. Concanavalin A depicted an aerobic profile of metabolism, once again confirming the improvement in OXPHOS.

The ATP Rate Index results (the ratio between the rate of mitoATP production and the rate of glyco-ATP production) showed that the values were approximately equal to 1 when no adhesion agents were used and in the presence of fibronectin, demonstrating the same amount of ATP produced by both metabolic pathways, i.e., mitochondrial respiration and glycolysis. The Matrigel^®^ matrix showed a value lower than 1, highlighting the greater contribution of glycolysis relative to cellular respiration in ATP production. Only in the presence of concanavalin A, in which the switch was towards an oxidative metabolism with ATP production by exploiting mitochondria, although there was no statistical significance between the various conditions (Figure 2d) (*p* > 0.05).

### 3.3. ATP Production After COCs Maturation

**Figure 3:**
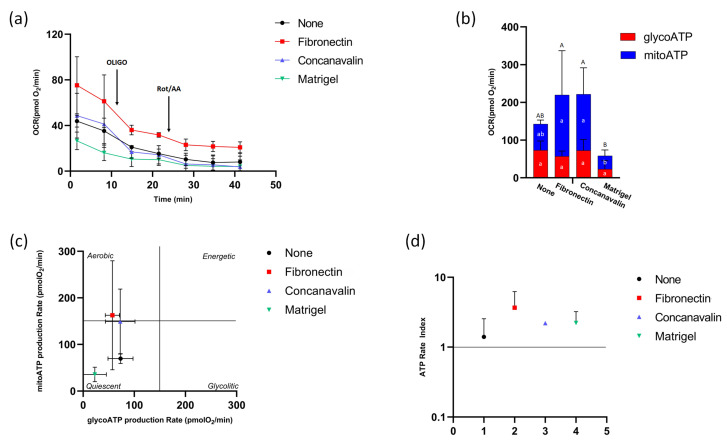
ATP production in mature COCs. (**a**) Metabolic profile obtained from oxygen consumption rate (OCR) in mature COCs, without (None) and with fibronectine, concanavalin A and Matrigel^®^ under basal respiration conditions and after addition of 1.5 μM oligomycin (olig), and a mixture of 0.5 μM rotenone plus antimycin A (Rot/AA). Modulator injections are shown with arrows. (**b**) Evaluation of ATP production rate by mitochondrial OXPHOS (blue, mitoATP) or by glycolysis (red, glycoATP) in the different conditions. (**c**) Energy map of mitoATP Production Rate vs. glycoATP Production Rate in mature COCs without (black) and with fibronectine (red), concanavalin A (blue) and Matrigel^®^ (green). (**d**) The ratio between the mitochondrial ATP production rate and the glycolytic ATP production rate (ATP rate index), is shown on the *y*-axis (logarithmic scale) in immature COCs under different conditions. Identical letters indicate no significant difference, whereas different letters indicate significant differences (*p* ≤ 0.05) among treatments within the same parameter (total ATP production, mitoATP, and glicoATP), respectively.

The metabolic profile of mature COC cells, assessed as for immature cells and shown in Figure 3a, showed that, after maturation, COC cells responded to the inhibitor oligomycin in all experimental conditions, although the best response was recorded in the presence of concanavalin A.

The rate of mitoATP and glycoATP production showed that, under all experimental conditions, mature COC cells relied primarily on mitochondrial oxidative metabolism to produce energy in the form of ATP (Figure 3b). However, Matrigel^®^ significantly reduced the amount of mitoATP compared to other conditions in the presence of adhesion agents (*p* < 0.05). The amount of total ATP also appeared significantly reduced when COC cells were adhered to Matrigel^®^ (*p* < 0.05), while it remained unchanged regardless of the use of adhesion agents compared to the control condition. The metabolic state of mature COCs (Figure 3c) was quiescent without and with Matrigel^®^, while fibronectin and concanavalin A allowed for more aerobic metabolism, confirming the increase in OXPHOS. The ATP Rate Index showed values > 1 in all conditions, confirming the predilection of a mitochondrial oxidative metabolism (Figure 3d).

## 4. Discussion

This study carried out on immature and mature bovine COCs allowed us to identify a metabolic characterization set on different adhesion conditions to the cell culture plate in order to exploit the Seahorse technology as a method for the energy metabolism analysis of bovine COCs.

Since it has been demonstrated that concanavalin A can interfere with the meiosis of mouse, goat, and mollusc oocytes [9,27,28,29], its possible effects on IVM were tested. One hour exposure of oocytes to concanavalin A at the beginning of IVM showed its inability to interfere with meiotic maturation. Immature COCs exhibit a predominantly glycolytic metabolic profile, confirmed not only by the higher proportion of glycolytic ATP in most conditions tested but also by the inability to respond to the modulator oligomycin. Although mitochondria are functional, responding to the inhibitors rotenone/antimycin A, they are not the primary source of ATP synthesis in the immature stage. This observation is consistent with a previous study showing that, at the onset of maturation, the majority of glucose uptake was directed into glycolysis. An anaerobic process might support energy production at this stage through ATP synthesis. However, by the end of the maturation period, a significant fraction of glucose appeared to be redirected into alternative metabolic pathways, possibly contributing to the synthesis of cumulus-cell extracellular matrix components [9]. Only in the presence of concanavalin A, immature COCs shifted to a more oxidative metabolism, producing a greater amount of mitoATP, likely due to concanavalin’s stimulatory effect on metabolism [27]. Concanavalin A promoted modest mitochondrial activation, confirmed by a metabolic state tending toward aerobic metabolism and an ATP Rate Index greater than 1.

There are no reports describing the use of concanavalin A as a coating for oocytes in Seahorse assays. However, in Seahorse-based studies of spermatozoa, concanavalin A has been successfully used to ensure strong and stable adhesion of sperm cells to the plates, thereby allowing accurate OCR and ECAR measurements without cell loss during mixing cycles [28,29,30,31]. Concanavalin A could support oxidative metabolism, potentially through enhanced mitochondrial function. At the same time, concanavalin-mediated adhesion may simply promote tighter attachment of immature COCs to the plate, ensuring accurate acquisition of OCR parameters.

Unlike immature cells, the maturation process allows for a shift in metabolism from glycolytic to oxidative [32], as confirmed by a positive response to oligomycin. This result is consistent with how mitochondria, through the production of energy and other essential components, support oocyte maturation, fertilization, and early embryonic development [33]. During oocyte maturation a higher energy supply is required, leading to a shift from glycolysis to oxidative phosphorylation [34]. Oxidative activity in bovine oocytes increases during maturation, particularly after 15 and 22 h of culture. This rise in oxidative metabolism coincides with key meiotic events of maturation, such as the formation of the metaphase plate during the first meiotic division and the extrusion of the first polar body [35]. Moreover, protein synthesis increases approximately threefold at the MI stage compared with the GV stage, suggesting that the enhanced oxidative activity observed after 15 h of maturation may be linked to the elevated demand for new protein synthesis [35].

Oocyte maturation is tightly regulated by endocrine and paracrine signals that directly modulate the metabolic activity of the COCs. Importantly, the maturation medium used in this study contained key hormones and growth factors, including FSH, LH, EGF, and IGF-1, which are known to stimulate meiotic resumption, cumulus expansion, and metabolic activation in the oocyte. These factors likely contributed to the observed shift from a predominantly glycolytic metabolism in immature COCs toward increased oxidative phosphorylation in mature COCs, highlighting the close interplay between endocrine/paracrine signalling and bioenergetic remodelling during IVM. The first step of meiotic resumption occurs by gonadotropins activating the surrounding GCs. FSH supports follicular growth, while LH induces meiotic progression and stimulates granulosa and cumulus cells to produce EGF-like peptides that activate EGFR and the MAPK/ERK pathway, promoting cumulus expansion and oocyte maturation [36]. This signaling increases cAMP in granulosa cells but reduces cAMP and cGMP in the oocyte by limiting gap junction communication, thereby triggering meiosis [37]. Fibronection, as with concanvalin A, appeared to provide the optimal cellular bioenergetics, promoting higher mitochondrial ATP production relative to total ATP generation. In the mature state, cellular energy demands are supported by OXPHOS, as indicated by an ATP Rate Index consistently greater than 1 and by a typical aerobic metabolic profile, particularly when assays were performed in the presence of concanavalin A and fibronectin.

In previous studies, fibronectin has been used as a coating substrate for Seahorse XF plates to promote cell adhesion during metabolic flux analysis. In particular, fibronectin-coated plates were employed to support the attachment of mouse granulosa cells and COCs during OCR measurements, allowing reliable metabolic assessment [38]. Similarly, fibronectin coating has been shown in other cellular models, such as spermatozoa, to enhance adhesion without interfering with mitochondrial or glycolytic function [23].

Matrigel^®^ has been previously employed in Seahorse assays with various cell types [39], but has never been used as a coating substrate for oocytes. In our experiments, Matrigel^®^ coating provided improved adhesion and yielded clearer metabolic signals in immature oocytes, highlighting their glycolytic activity. Unlike when the COCs were stabilized on the bottom, the Matrigel^®^ matrix rendered the metabolism of mature COCs quiescent, as occurs even without the adhesion agent, suggesting that incorrect/lack of adhesion to the plate altered the analysis. Moreover, the metabolic quiescence observed in mature COCs on Matrigel^®^ may reflect the three-dimensional matrix that can impair firm immobilization during Seahorse mixing cycles and increase diffusion distances that dilute local oxygen and proton gradients, thereby reducing apparent OCR/ECAR. In addition, Matrigel’s complex composition and variable growth-factor content may alter integrin-mediated signaling, cytoskeletal tension, and cumulus cell differentiation, potentially contributing to genuine shifts in substrate utilization and mitochondrial activity, so both mechanical positioning and biological cueing should be considered [40].

The absence of a coating deserves careful consideration. Previous studies assessing COC metabolism with the Seahorse Analyzer performed measurements using uncoated wells, reporting stable and interpretable metabolic activity [41,42]. In contrast, under our experimental conditions, both immature and mature oocytes exhibited a markedly quiescent metabolic profile when analyzed in uncoated wells. This discrepancy suggests that the absence of a coating may impair the interaction between the oocyte–cumulus complex and the plate surface, potentially limiting proper cell attachment or stabilization during mixing cycles and thereby affecting OCR and ECAR detection. It is also plausible that differences in microplate design, surface charge, or material composition between manufacturers, or even between production batches, contribute to the divergent outcomes reported across studies. Such variability underscores the importance of carefully evaluating plate–cell interactions and supports the use of adhesion-promoting coatings to ensure reliable and biologically meaningful metabolic measurements.

This study focused on in vitro matured COCs, which are known to exhibit lower developmental competence compared to in vivo matured counterparts. Current IVM systems does not fully recapitulate the in vivo environment and IVM oocytes exhibit lower developmental competence, partly due to suboptimal culture conditions that fail to adequately support the metabolic requirements of the COCs, resulting in altered metabolic profiles compared to in vivo matured counterparts, including reduced glucose metabolism, impaired redox balance, lower ATP/ADP ratios, and lipid accumulation [17]. While direct comparisons with in vivo matured COCs were beyond the scope of this study, such analyses would be valuable for identifying specific metabolic deviations induced by IVM and for optimizing culture conditions. Although this study was conducted on bovine oocytes, species-specific differences in oocyte metabolism should be considered when extrapolating the results. Expanding the analyses to other species, including human and mouse oocytes, may help assess the broader applicability of these findings and identify conserved metabolic markers of oocyte competence. Standardized Seahorse-based bioenergetic assessments could support the optimization of IVM protocols, improve embryo selection, and ultimately enhance translational applications in assisted reproductive technologies.

**Figure 4:**
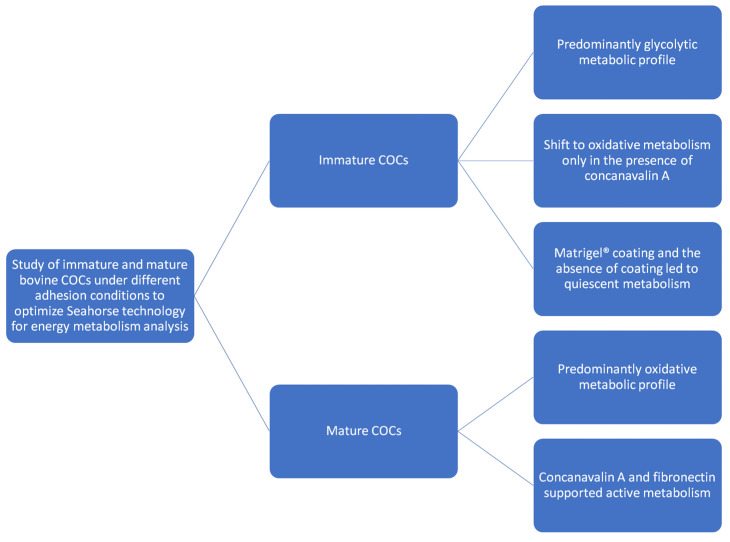
Overview of the bioenergetic map in immature and mature bovine COCs.

On balance, cell metabolism plays a decisive role in supporting oocyte maturation, regulating ATP availability, and the metabolic plasticity required for successful developmental competence. In this context, the bioenergetic profiles of immature and mature bovine COCs offer valuable insight into how mitochondrial/glycolytic activity adapts throughout the maturation process (Figure 4).

## 5. Conclusions

In conclusion, this study demonstrates that Seahorse XFp technology can be effectively applied to characterize the bioenergetic profile of bovine cumulus–oocyte complexes and to discriminate metabolic states associated with maturation. Under the conditions employed in the studies, we identified HSOF as the most suitable medium for immature COCs and TCM199 for mature COCs, highlighting the importance of tailoring assay conditions to the developmental stage. Using real-time measurements of OCR and ECAR together with mitochondrial inhibitors, we show that maturation is accompanied by a clear metabolic transition from a predominantly glycolytic phenotype in immature COCs toward increased oxidative metabolism and active mitochondrial ATP production after 22 h of IVM. Importantly, adhesion conditions strongly affected the recorded metabolic output: concanavalin A and fibronectin supported reliable attachment and were consistently associated with robust energetic profiles, whereas poor adhesion, particularly in Matrigel^®^, was linked to quiescent metabolism, suggesting an artefact driven by inadequate adherence rather than true metabolic suppression

Collectively, these findings establish a standardized workflow for Seahorse-based assessment of COC bioenergetics and underscore the central contribution of mitochondrial function to oocyte maturation. This framework provides a practical basis for future studies and may support the refinement of in vitro maturation strategies through informed modulation of culture conditions.

## Figures and Tables

**Table 1:** Maturation rate (mean ± SD) of bovine COCs cultured in basal maturation medium for 22 h, without (CTR) or with exposure to concanavalin A (ConA; 0.39 mg/mL) for 1 h at the beginning of the IVM.

Group	Stained Oocytes (n)	Mature Oocytes (%)	Immature Oocytes (%)	Degenerate Oocytes (%)
CTR	86	65.9 ± 9.7	25.8 ± 7.9	8.3 ± 2.8
ConA	86	59.5 ± 2.6	33.4 ± 3.6	7.1 ± 1.1

## Data Availability

The data that support the findings of this study are openly available on the AMSActa Institutional Research Repository by AlmaDL University of Bologna Digital Library https://amsacta.unibo.it/id/eprint/8730, accessed on 23 January 2026.
